# Supplementary material for: Adipocytic sclerostin loop3-LRP4 interaction required by sclerostin to impair whole-body lipid and glucose metabolism
Source: Nat Commun. 2026 Jan 16;17:1812. doi: 10.1038/s41467-026-68526-w (PMC12917164; doi:10.1038/s41467-026-68526-w)
Supplement: Supplementary file 1 — Supplementary Information [file 41467_2026_68526_MOESM1_ESM.pdf]

Adipocytic sclerostin loop3-LRP4 interaction required by sclerostin to impair whole-body lipid and glucose metabolism

Hewen Jiang [1#], Xiaohui Tao [2,3,4#], Sifan Yu [2,3,4#], Yihao Zhang [1#], Yuan Ma [1#], Nanxi Li [2,3,4], Shenghang Wang [2,3,4], Ning Zhang [1], Xin Yang [2,3,4], Shijian Ding [2,3,4], Chuanxin Zhong [2,3,4], Haitian Li [2,3,4], Zhanghao Li [2,3,4], Xiaoxin Wen [2,3,4], Huarui Zhang [1], Zefeng Chen [2,3,4], Meiheng Sun [2,3,4], Hang Luo [1], Meishen Ren [2,3,4], Chongguang Lei [1], Yuanyuan Yu [2,3,4], Jin Liu [2,3,4], Zongkang Zhang [1], Aiping Lyu [2,3,4], Hui Sheng [5*], Dijie Li [2,3,4,6,7,8,9*], Luyao Wang [2,3,4*], Ge Zhang [2,3,4*], Bao-Ting Zhang [1*].

[1] School of Chinese Medicine, The Chinese University of Hong Kong, Hong Kong, China.
[2] Guangdong-Hong Kong Macao Greater Bay Area International Research Platform for Aptamer-Based Translational Medicine and Drug Discovery, Hong Kong, China.
[3] Law Sau Fai Institute for Advancing Translational Medicine in Bone & Joint Diseases, School of Chinese Medicine, Hong Kong Baptist University, Hong Kong, China.
[4] Institute of Integrated Bioinformedicine and Translational Science, School of Chinese Medicine, Hong Kong Baptist University, Hong Kong, China.
[5] Osteoporosis and Sarcopenia Center, Department of Endocrinology and Metabolism, School of Medicine, Tongji University, The Shanghai Tenth People's Hospital, Shanghai, China.
[6] Guangxi Universities Key Laboratory of Stem cell and Biopharmaceutical Technology, College of Life Sciences, Guangxi Normal University, Guilin, 541004, Guangxi, China.
[7] Shenzhen Institute for Research and Continuing Education (IRACE), Hong Kong Baptist University, Shenzhen, 518057, Guangdong, China.
[8] Research Center for Biomedical Sciences, Guangxi Normal University, Guilin, 541004, Guangxi, China.
[9] Key Laboratory of Ecology of Rare and Endangered Species and Environmental Protection (Ministry of Education), Guangxi Normal University, Guilin, 541004, Guangxi, China.

[#] These authors contributed equally to this study.
[*] Corresponding author: Dr. Dijie Li (lidijie@gxnu.edu.cn), Dr. Luyao Wang (luyaowang@hkbu.edu.hk), Prof. Hui Sheng (shenghui@tongji.edu.cn), Prof. Ge Zhang (zhangge@hkbu.edu.hk), Prof. Bao-Ting Zhang (zhangbaoting@cuhk.edu.hk).

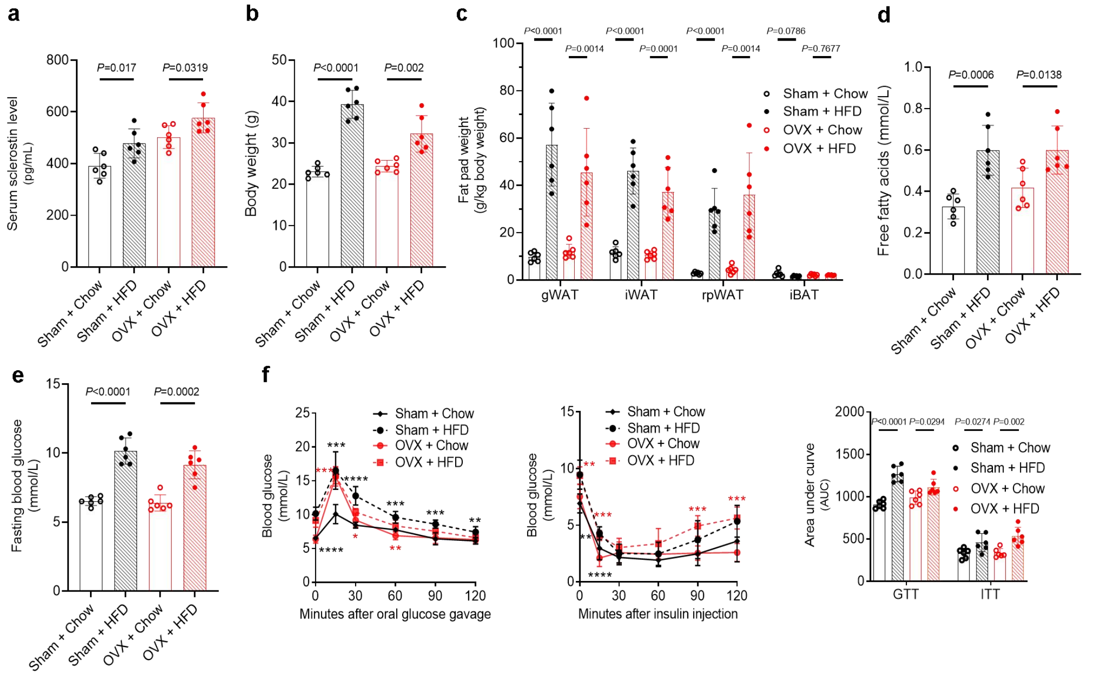

Supplementary Figure 1. Circulating sclerostin levels were significantly higher in ovariectomized (OVX) mice with high-fat diet induction (OVX + HFD) compared to OVX mice with chow diet (OVX + Chow). (a) Serum sclerostin levels in Sham and OVX mice fed with chow diet or HFD. (b) Body weights in Sham and OVX mice fed with chow diet or HFD. (c) Fat pad weights in Sham and OVX mice fed with chow diet or HFD. (d) Serum free fatty acids in Sham and OVX mice fed with chow diet or HFD. (e) Fasting blood glucose in Sham and OVX mice fed with chow diet or HFD. (f) Glucose tolerance test (GTT) (left), insulin tolerance test (ITT) (middle), and area under the curve (AUC) analysis for GTT and ITT (right) in Sham and OVX mice fed with chow diet or HFD. Note: n = 6 biologically independent samples per group. All data were expressed as mean ± SD. **P* < 0.05, ***P* < 0.01, ****P* < 0.001 and *****P* < 0.0001 for intergroup comparison (black *: Sham + Chow vs. Sham + HFD; red *: OVX + Chow vs. OVX + HFD) by unpaired t-test. ns: no significance. All tests were two-sided.

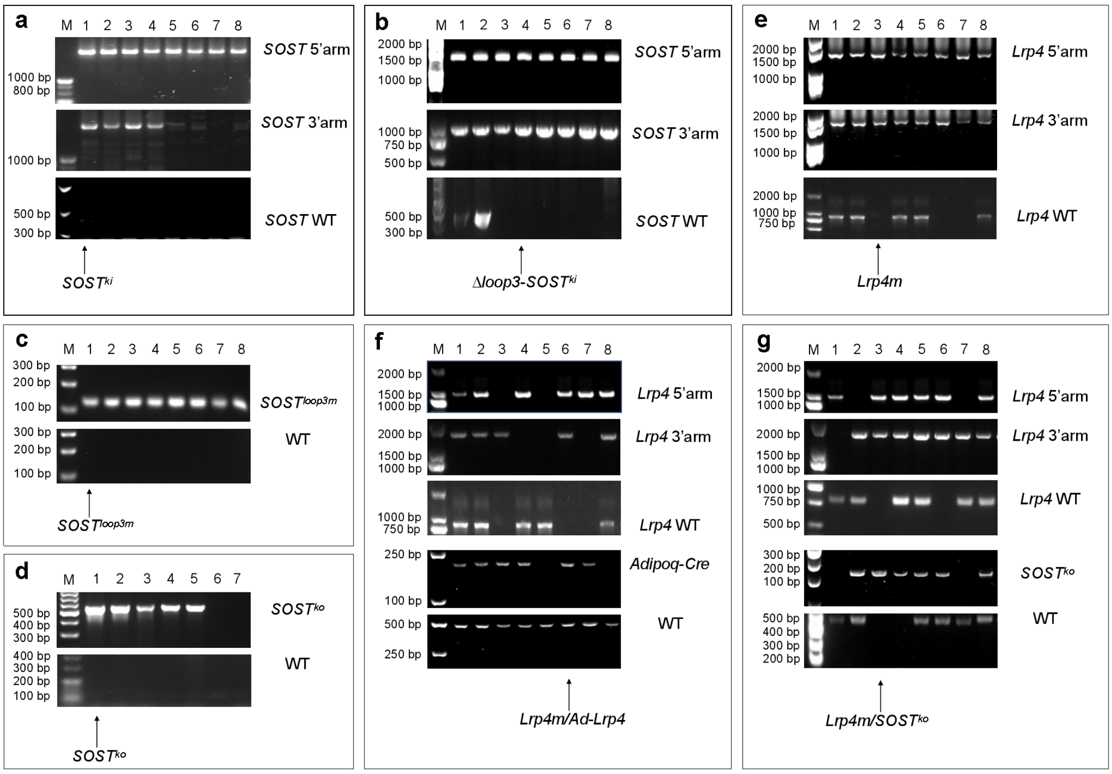

Supplementary Figure 2. Genotyping of $SOST^{ki}$ mice, Δ*loop3-*$SOST^{ki}$ mice, $SOST^{loop3m}$ mice, $SOST^{ko}$ mice, *Lrp4m* mice, *Lrp4m/Ad-Lrp4* mice and *Lrp4m/*$SOST^{ko}$ mice. (a) Representative agarose gel electrophoretic images for PCR genotyping samples from $SOST^{ki}$ mice. (b) Representative agarose gel electrophoretic images for PCR genotyping samples from Δ*loop3-*$SOST^{ki}$ mice. (c) Representative agarose gel electrophoretic images for PCR genotyping samples from $SOST^{loop3m}$ mice. (d) Representative agarose gel electrophoretic images for PCR genotyping samples from $SOST^{ko}$ mice. (e) Representative agarose gel electrophoretic images for PCR genotyping samples from *Lrp4m* mice. (f) Representative agarose gel electrophoretic images for PCR genotyping samples from *Lrp4m/Ad-Lrp4* mice. (g) Representative agarose gel electrophoretic images for PCR genotyping samples from *Lrp4m/*$SOST^{ko}$ mice. Note: $SOST^{ki}$ mice: 5'arm～1465 bp, 3'arm～1229 bp, wild-type (WT)～412 bp; Δ*loop3-*$SOST^{ki}$ mice: 5'arm～1465 bp, 3'arm～825 bp, WT～412 bp; $SOST^{loop3m}$ mice: $SOST^{loop3m}$～121 bp, WT～398 bp; $SOST^{ko}$ mice: $SOST^{ko}$～555 bp, WT～378 bp. *Lrp4m* mice: 5'arm～1567 bp, 3'arm～1929 bp, WT～835 bp. *Lrp4m/Ad-Lrp4* mice: *Lrp4m* (5'arm～1567 bp, 3'arm ～ 1929 bp, WT ～ 835 bp), Adipoq-Cre (5'arm ～ 200 bp, 3'arm ～ 507 bp). *Lrp4m/*$SOST^{ko}$ mice: *Lrp4m* (5'arm～1567 bp, 3'arm～1929 bp, WT～835 bp), $SOST^{ko}$ ($SOST^{ko}$ ～ 555 bp, WT ～ 378 bp). In homozygous mice, the WT band could not be detected. All tests were two-sided.

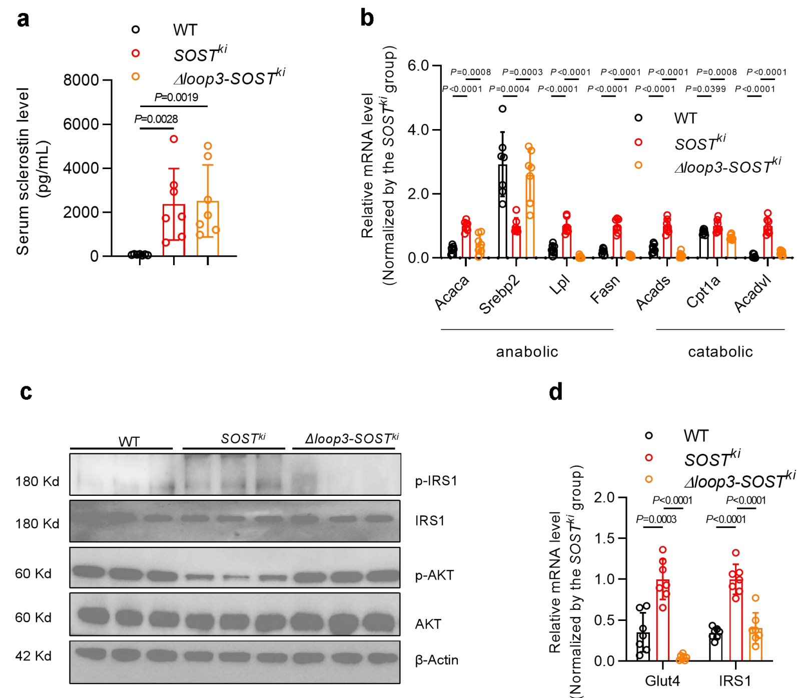

Supplementary Figure 3. The serum sclerostin levels and expression levels of genes associated with lipid and glucose metabolism in iWAT and phosphorylation levels of IRS1 and AKT in gWAT from wild-type (WT) mice, *SOST^{ki}* mice and Δ*loop3-SOST^{ki}* mice. (a) Serum sclerostin levels in WT mice, *SOST^{ki}* mice and Δ*loop3-SOST^{ki}* mice. (b) Expression levels of genes associated with lipid anabolism (*Acaca*, *Srebp2*, *Lpl* and *Fasn*) and catabolism (*Acads*, *Cpt1a* and *Acadvl*) in iWAT from WT mice, *SOST^{ki}* mice and Δ*loop3-SOST^{ki}* mice detected by qPCR. (c) Immunoblotting of phosphorylated IRS1 and AKT in gWAT from WT mice, *SOST^{ki}* mice and Δ*loop3-SOST^{ki}* mice. (d) Expression levels of genes associated with glucose metabolism in iWAT from WT mice, *SOST^{ki}* mice and Δ*loop3-SOST^{ki}* mice detected by qPCR. Note: n = 7 biologically independent samples for serum sclerostin level and qPCR analysis. n = 3 biologically independent samples for western blot analysis. Statistical significance was calculated using unpaired t-test. All tests were two-sided.

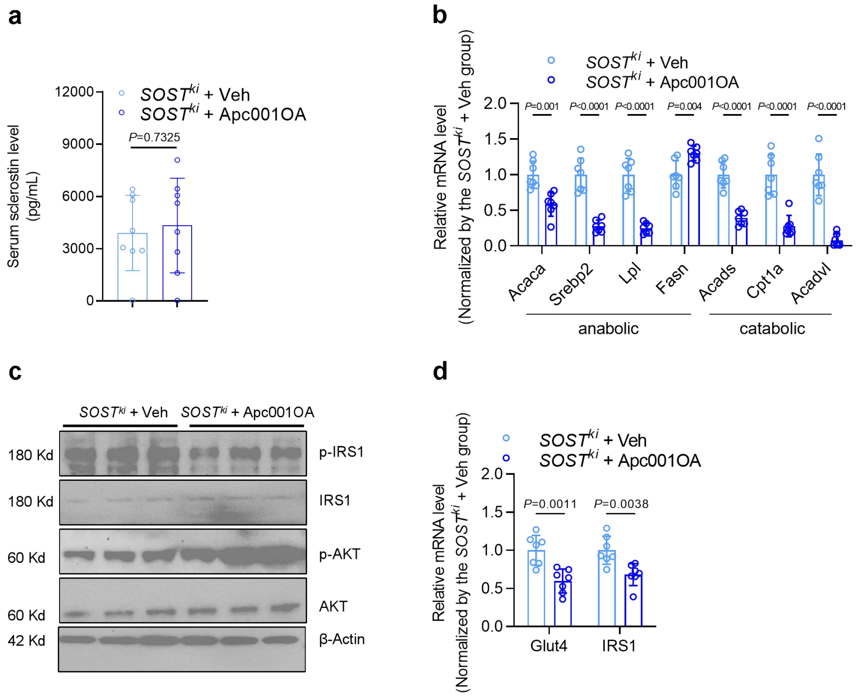

Supplementary Figure 4. The serum sclerostin levels and expression levels of genes associated with lipid and glucose metabolism in iWAT and phosphorylation levels of IRS1 and AKT in gWAT from *SOST^{ki}* mice with or without Apc001OA treatment. (a) Serum sclerostin levels in *SOST^{ki}* mice with or without Apc001OA treatment. (b) Expression levels of genes associated with lipid anabolism (*Acaca*, *Srebp2*, *Lpl* and *Fasn*) and catabolism (*Acads*, *Cpt1a* and *Acadvl*) in iWAT from *SOST^{ki}* mice with or without Apc001OA treatment detected by qPCR. (c) Immunoblotting of phosphorylated IRS1 and AKT in gWAT from *SOST^{ki}* mice with or without Apc001OA treatment. (d) Expression levels of genes associated with glucose metabolism in iWAT from *SOST^{ki}* mice with or without Apc001OA treatment detected by qPCR. Note: n = 7 biologically independent samples for serum sclerostin level and qPCR analysis. n = 3 biologically independent samples for western blot analysis. Statistical significance was calculated using unpaired t-test. ns: no significance. All tests were two-sided.

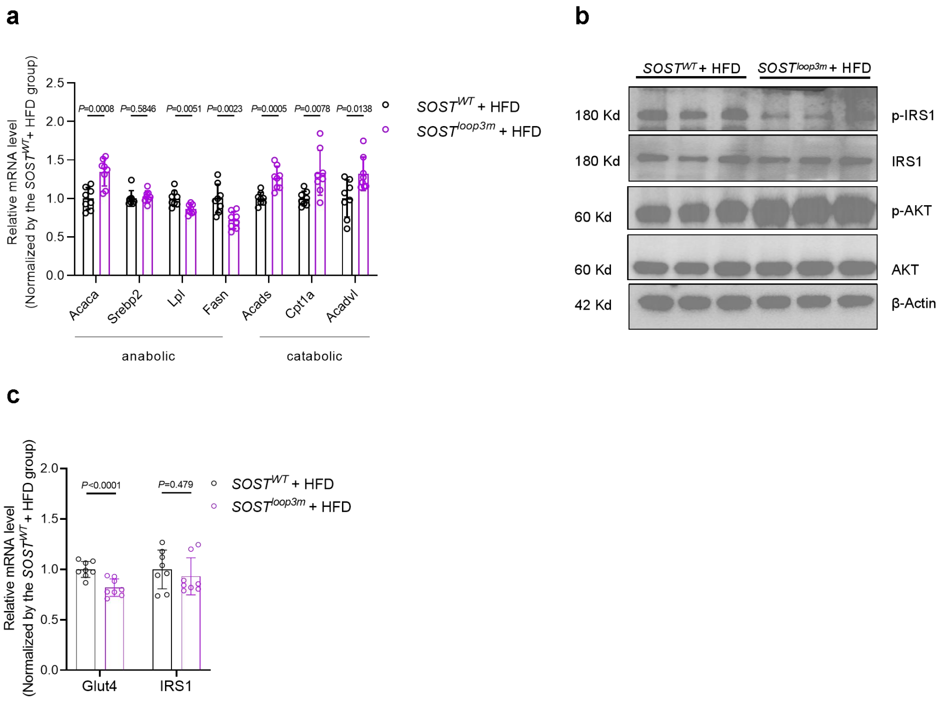

Supplementary Figure 5. The expression levels of genes associated with lipid and glucose metabolism in iWAT and phosphorylation levels of IRS1 and AKT in gWAT from *SOST*^{loop3m} + HFD mice and *SOST*^{WT} + HFD mice. (a) Expression levels of genes associated with lipid anabolism (*Acaca*, *Srebp2*, *Lpl* and *Fasn*) and catabolism (*Acads*, *Cpt1a* and *Acadvl*) in iWAT from *SOST*^{loop3m} + HFD mice and *SOST*^{WT} + HFD mice detected by qPCR. (b) Immunoblotting of phosphorylated IRS1 and AKT in gWAT from *SOST*^{loop3m} + HFD mice and *SOST*^{WT} + HFD mice. (c) Expression levels of genes associated with glucose metabolism in iWAT from *SOST*^{loop3m} + HFD mice and *SOST*^{WT} + HFD mice detected by qPCR. Note: n = 8 biologically independent samples for qPCR analysis. n = 3 biologically independent samples for western blot analysis. Statistical significance was calculated using unpaired t-test. ns: no significance. All tests were two-sided.

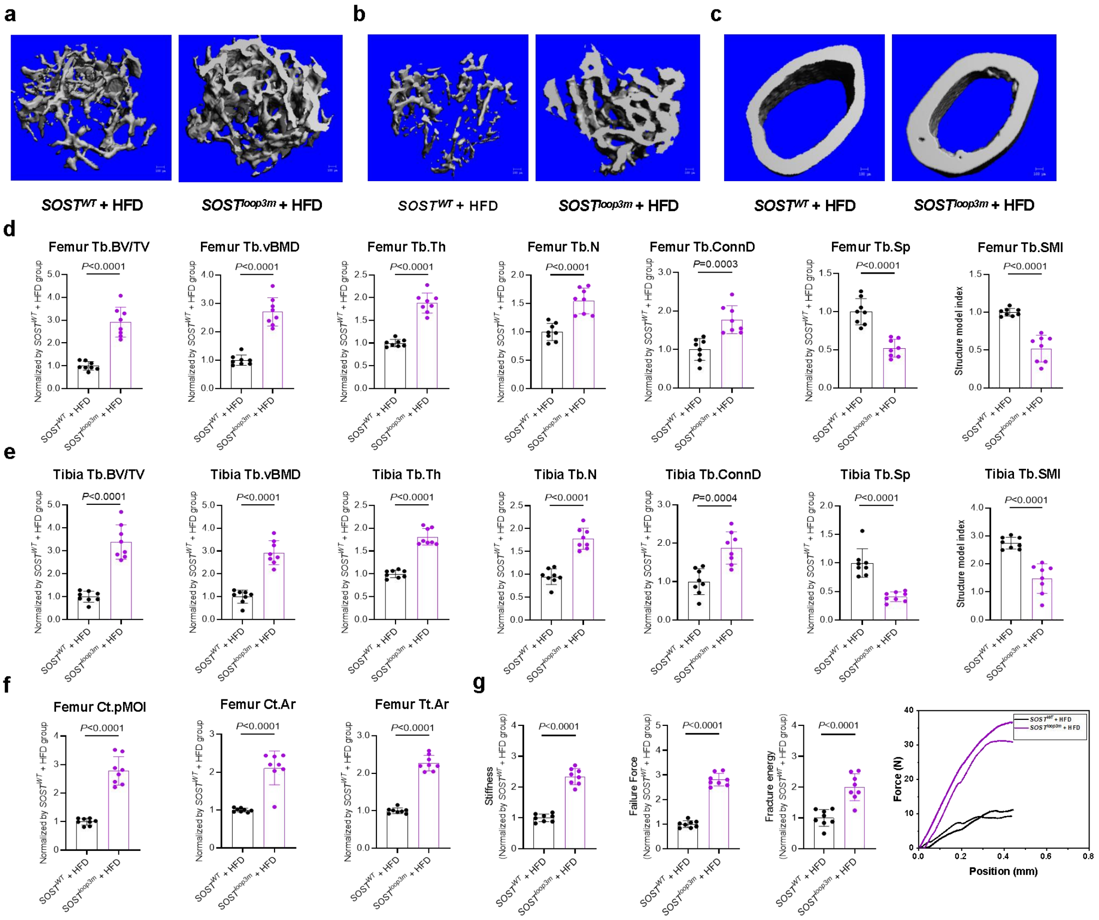

Supplementary Figure 6. The bone mass, bone microarchitecture and mechanical properties were improved by loop3 mutation in *SOST*^{loop3m} + HFD mice compared to those in *SOST*^{WT} + HFD mice. (a) Representative images showing three-dimensional trabecular bone microarchitecture at the distal femur. Scale bars, 100 μm. (b) Representative images showing three-dimensional trabecular bone microarchitecture at the proximal tibia. Scale bars, 100 μm. (c) Representative images showing three-dimensional trabecular bone microarchitecture at the femoral mid-shaft. Scale bars, 100 μm. (d) Bar charts of the structural parameters of Tb.BV/TV, Tb.vBMD, Tb.Th, Tb.N, Tb.Conn.D, Tb.Sp and Tb.SMI at the distal femur. (e) Bar charts of the structural parameters of Tb.BV/TV, Tb.vBMD, Tb.Th, Tb.N, Tb.Conn.D, Tb.Sp and Tb.SMI at the proximal tibia. (f) Bar charts of the structural parameters of Ct.pMOI, Ct.Ar and Tt.Ar at the femoral mid-shaft. (g) Three-point bending test for the normalized failure force (left), stiffness (middle) and fracture energy (right) at the femoral mid-shaft. Note: n = 8 biologically independent samples per group. Tb.BV/TV: trabecular relative bone volume; Tb.vBMD: trabecular volumetric mineral density; Tb.Th: trabecular thickness; Tb.N: trabecular number; Tb.Conn.D: trabecular connection density; Tb.Sp: trabecular spacing; Tb.SMI: trabecular structure model index. Ct.pMOI: cortical polar moment of inertia; Ct.Ar: cortical bone area; Tt.Ar: total cortical area. Statistical significance was calculated using unpaired t-test. All tests were two-sided.

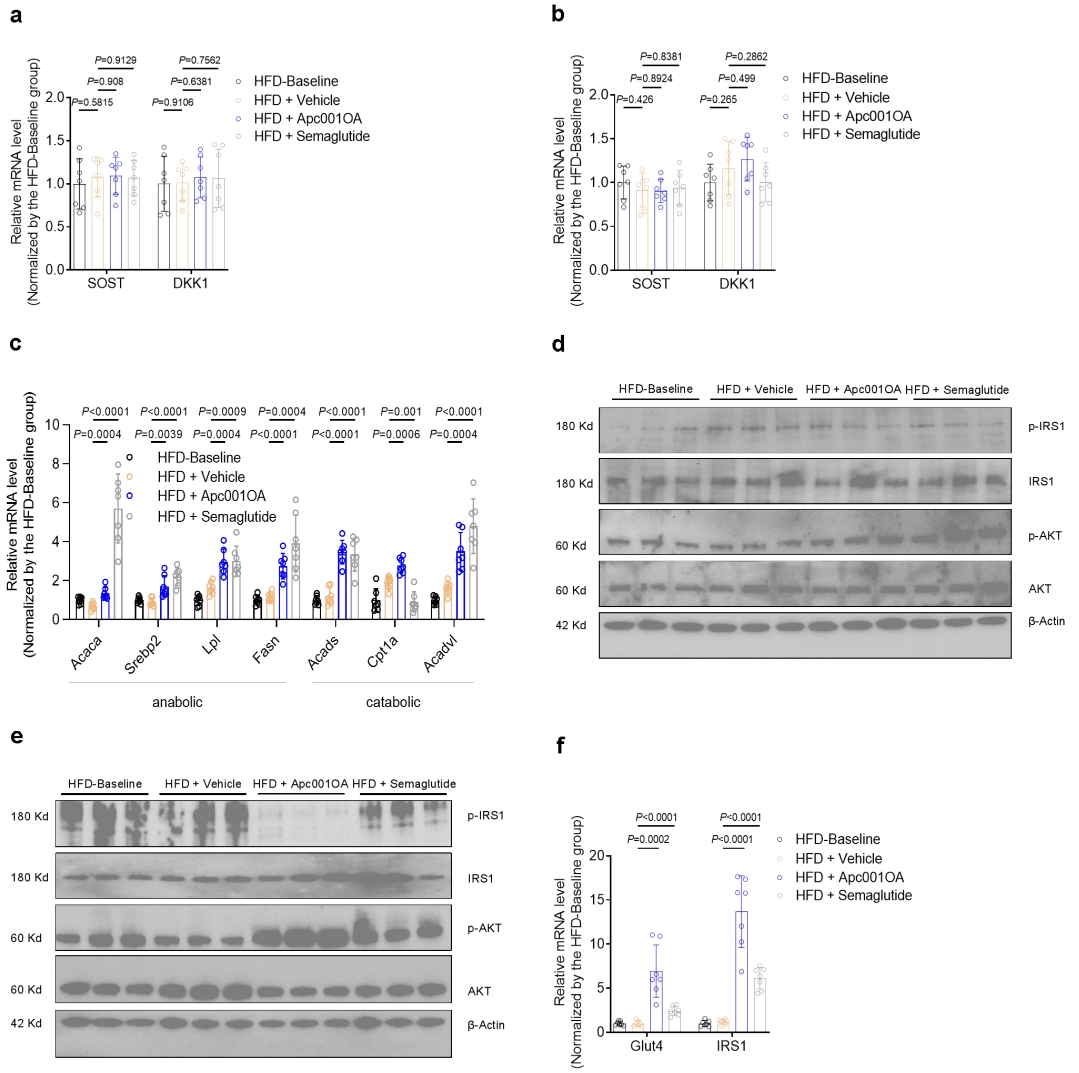

Supplementary Figure 7. The expression levels of *SOST* and *DKK1* in gWAT and iWAT, genes associated with lipid and glucose metabolism in iWAT and phosphorylation levels of IRS1 and AKT in gWAT from HFD-induced mice with or without Apc001OA treatment. (a) Expression levels of *SOST* and *DKK1* in gWAT from HFD-induced mice with or without Apc001OA treatment detected by qPCR. (b) Expression levels of *SOST* and *DKK1* in iWAT from HFD-induced mice with or without Apc001OA treatment detected by qPCR. (c) Expression levels of genes associated with lipid anabolism (*Acaca*, *Srebp2*, *Lpl* and *Fasn*) and catabolism (*Acads*, *Cpt1a* and *Acadvl*) in iWAT from HFD-induced mice with or without Apc001OA treatment detected by qPCR. (d) Immunoblotting of phosphorylated IRS1 and AKT in gWAT from HFD-induced mice with or without Apc001OA treatment. (e) Immunoblotting of phosphorylated IRS1 and AKT in gastrocnemius muscle from HFD-induced mice with or without Apc001OA treatment. (f) Expression levels of genes associated with glucose metabolism in iWAT from HFD-induced mice with or without Apc001OA treatment detected by qPCR. Note: n = 7 biologically independent samples for qPCR analysis. n = 3 biologically independent samples for western blot analysis. Statistical significance was calculated using unpaired t-test. All tests were two-sided.

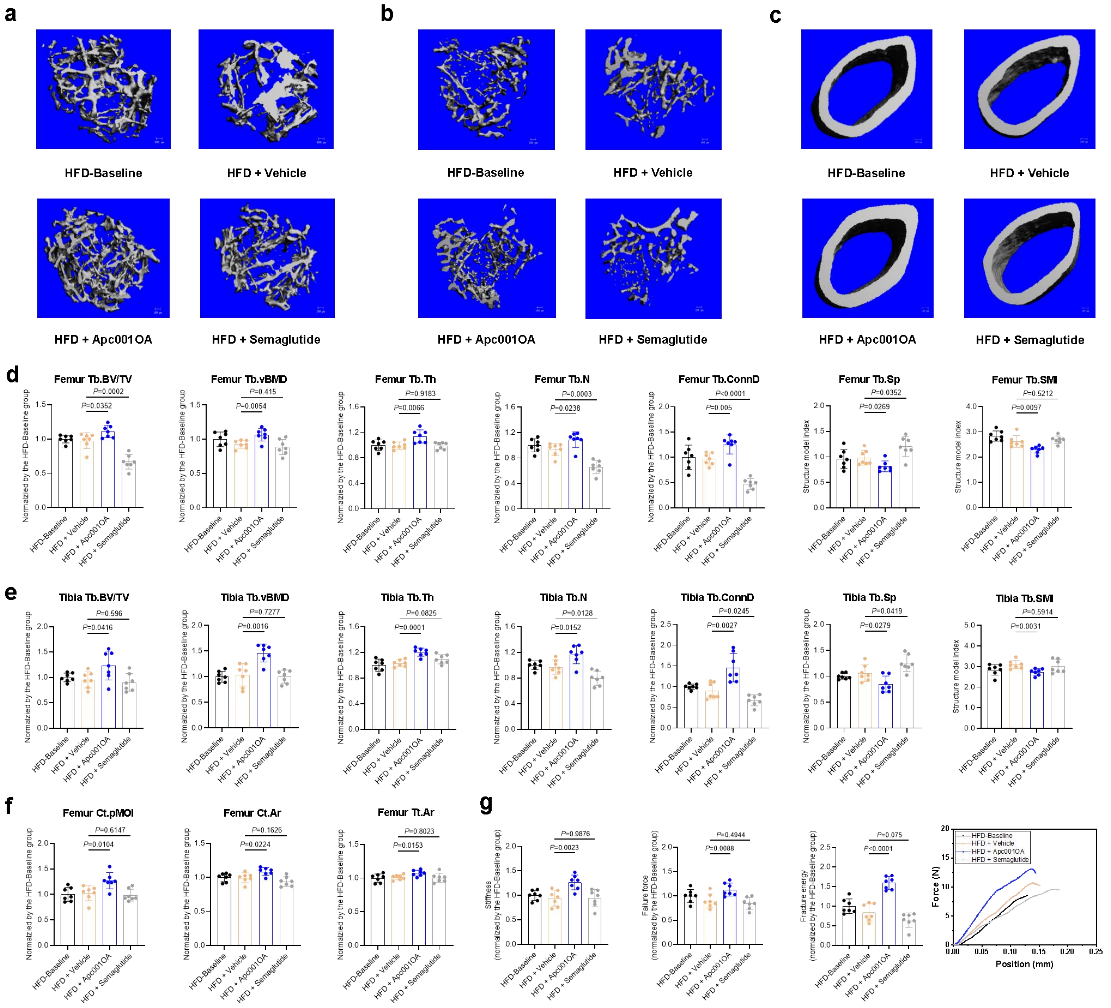

Supplementary Figure 8. The bone mass, bone microarchitecture and mechanical properties were improved in HFD-induced mice with Apc001OA treatment. (a) Representative images showing three-dimensional trabecular bone microarchitecture at the distal femur. Scale bars, 100 μm. (b) Representative images showing three-dimensional trabecular bone microarchitecture at the proximal tibia. Scale bars, 100 μm. (c) Representative images showing three-dimensional trabecular bone microarchitecture at the femoral mid-shaft. Scale bars, 100 μm. (d) Bar charts of the structural parameters of Tb.BV/TV, Tb.vBMD, Tb.Th, Tb.N, Tb.Conn.D, Tb.Sp and Tb.SMI at the distal femur. (e) Bar charts of the structural parameters of Tb.BV/TV, Tb.vBMD, Tb.Th, Tb.N, Tb.Conn.D, Tb.Sp and Tb.SMI at the proximal tibia. (f) Bar charts of the structural parameters of Ct.pMOI, Ct.Ar and Tt.Ar at the femoral mid-shaft. (g) Three-point bending test for the normalized failure force (left), stiffness (middle) and fracture energy (right) at the femoral mid-shaft. ns: no significance. Note: n = 7 biologically independent samples per group. Tb.BV/TV: trabecular relative bone volume; Tb.vBMD: trabecular volumetric mineral density; Tb.Th: trabecular thickness; Tb.N: trabecular number; Tb.Conn.D: trabecular connection density; Tb.Sp: trabecular spacing; Tb.SMI: trabecular structure model index. Ct.pMOI: cortical polar moment of inertia; Ct.Ar: cortical bone area; Tt.Ar: total cortical area. Statistical significance was calculated using unpaired t-test. All tests were two-sided.

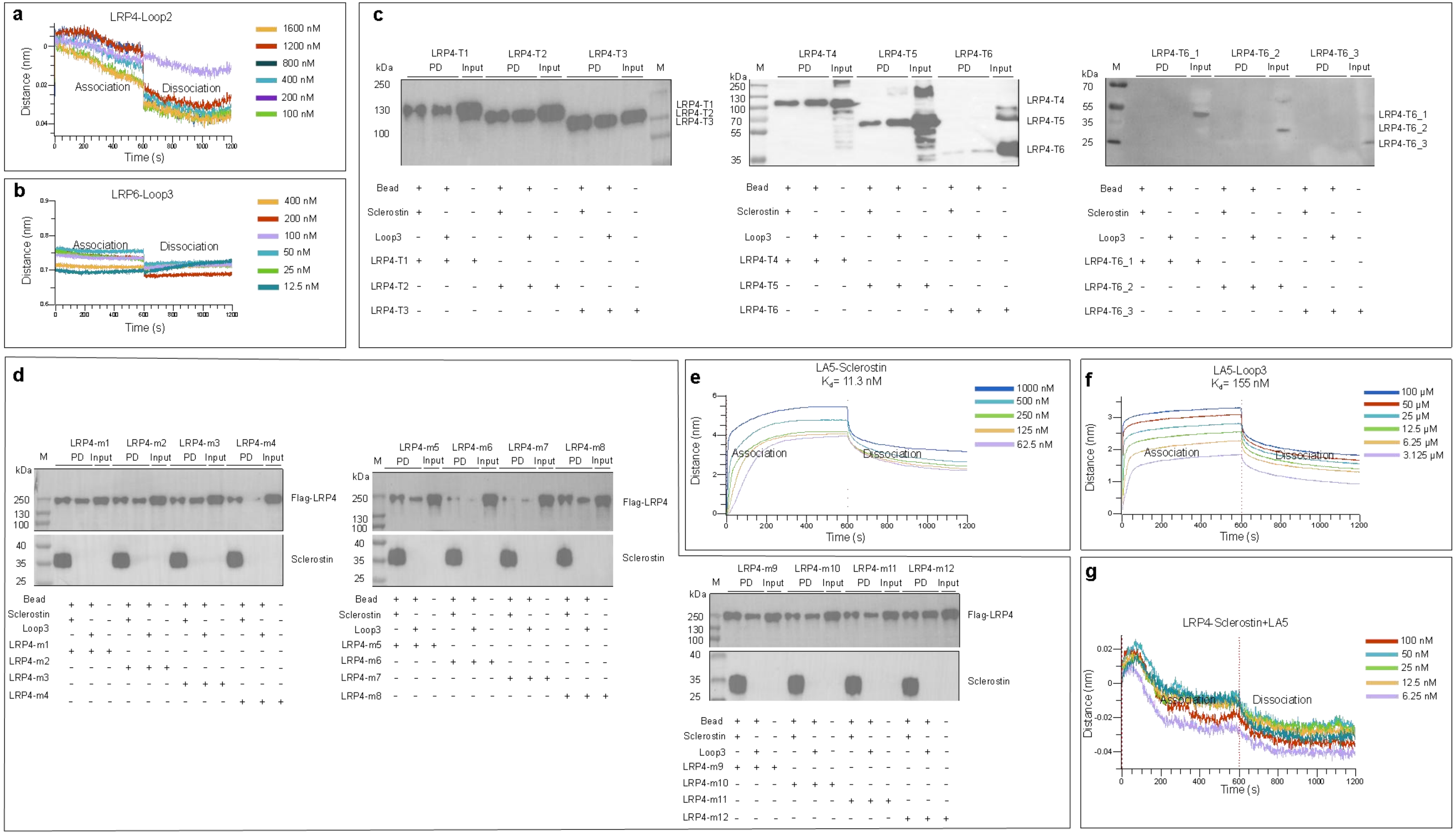
a
LRP4-Loop2
1600 nM
1200 nM
800 nM
400 nM
200 nM
100 nM
Distance (nm)
Association
Dissociation
Time (s)
b
LRP6-Loop3
400 nM
200 nM
100 nM
50 nM
25 nM
12.5 nM
Distance (nm)
Association
Dissociation
Time (s)
c
LRP4-T1
LRP4-T2
LRP4-T3
LRP4-T4
LRP4-T5
LRP4-T6
LRP4-T6_1
LRP4-T6_2
LRP4-T6_3
kDa
M
PD
Input
Bead
Sclerostin
Loop3
d
LRP4-m1
LRP4-m2
LRP4-m3
LRP4-m4
LRP4-m5
LRP4-m6
LRP4-m7
LRP4-m8
LRP4-m9
LRP4-m10
LRP4-m11
LRP4-m12
Flag-LRP4
Sclerostin
e
LA5-Sclerostin
K_d= 11.3 nM
1000 nM
500 nM
250 nM
125 nM
62.5 nM
Distance (nm)
Association
Dissociation
Time (s)
f
LA5-Loop3
K_d= 155 nM
100 μM
50 μM
25 μM
12.5 μM
6.25 μM
3.125 μM
Distance (nm)
Association
Dissociation
Time (s)
g
LRP4-Sclerostin+LA5
100 nM
50 nM
25 nM
12.5 nM
6.25 nM
Distance (nm)
Association
Dissociation
Time (s)

Supplementary Figure 9. The identification of binding residues within LRP4 to sclerostin loop3 and design of blocking peptide LA5 in adipocytes *in vitro*. (a) BLI analysis of the binding affinity between sclerostin loop2 and LRP4. (b) BLI analysis of the binding affinity between sclerostin loop3 and LRP6. (c) Binding ability of LRP4 truncations (LRP4 T1-T6, LRP4 T6_1, LRP4 T6_2, LRP4 T6_3) to sclerostin loop3 by pull-down assay for identifying the binding domains on LRP4 to sclerostin loop3. (d) Binding ability of LRP4 muteins (LRP4 m1-m12) to sclerostin loop3 by pull-down assay for identifying the binding residues within LRP4 to sclerostin loop3. (e) BLI analysis of the binding affinity between LA5 and sclerostin. (f) BLI analysis of the binding affinity between LA5 and sclerostin loop3. (g) BLI analysis of the binding affinity between LRP4 and sclerostin pre-incubated with LA5 . Note: BLI: biolayer interferometry; PD: pull-down.

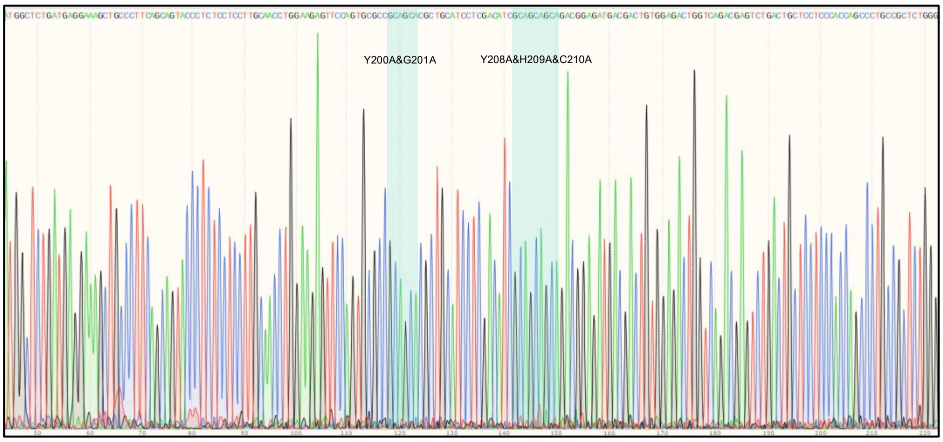

Supplementary Figure 10. The sequencing result of mutated sequence in *Lrp4m* mice. The mutated base sequences were marked in aqua.

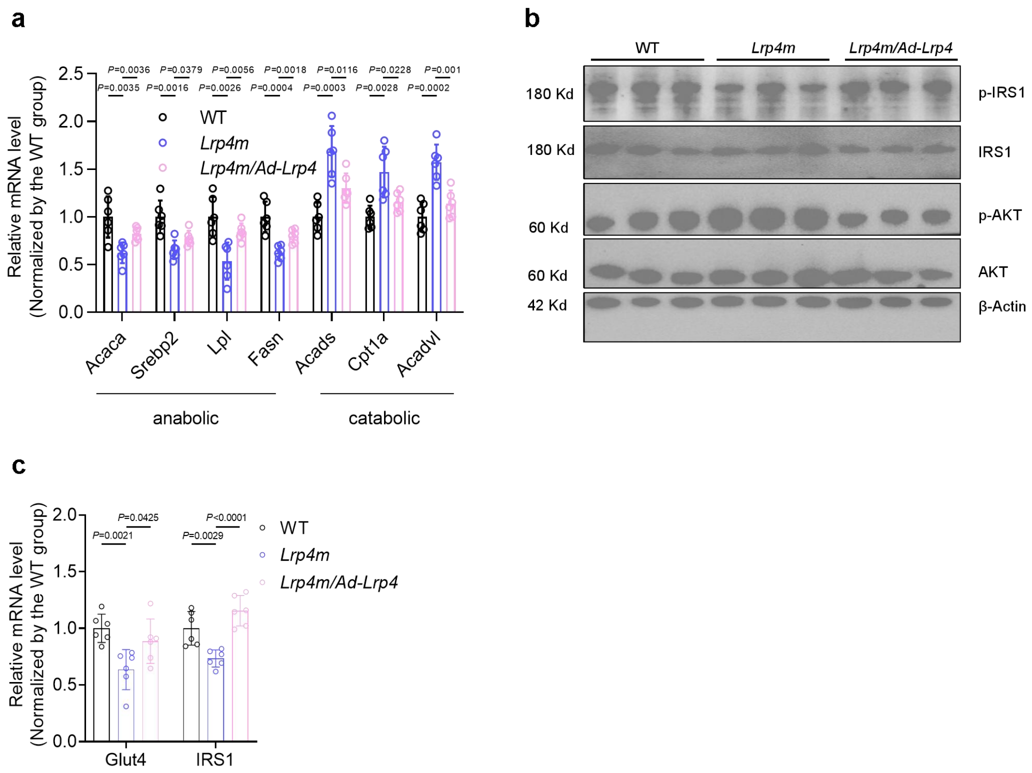

Supplementary Figure 11. The expression levels of genes associated with lipid and glucose metabolism in iWAT and phosphorylation levels of IRS1 and AKT in gWAT from wild-type (WT) mice, *Lrp4m* mice and *Lrp4m/Ad-Lrp4* mice. (a) Expression levels of genes associated with lipid anabolism (*Acaca*, *Srebp2*, *Lpl* and *Fasn*) and catabolism (*Acads*, *Cpt1a* and *Acadvl*) in iWAT from WT mice, *Lrp4m* mice and *Lrp4m/Ad-Lrp4* mice detected by qPCR. (b) Immunoblotting of phosphorylated IRS1 and AKT in gWAT from WT mice, *Lrp4m* mice and *Lrp4m/Ad-Lrp4* mice. (c) Expression levels of genes associated with glucose metabolism in iWAT from WT mice, *Lrp4m* mice and *Lrp4m/Ad-Lrp4* mice detected by qPCR. Note: n = 6 biologically independent samples for qPCR analysis. n = 3 biologically independent samples for western blot analysis. Statistical significance was calculated using unpaired t-test. ns: no significance. All tests were two-sided.

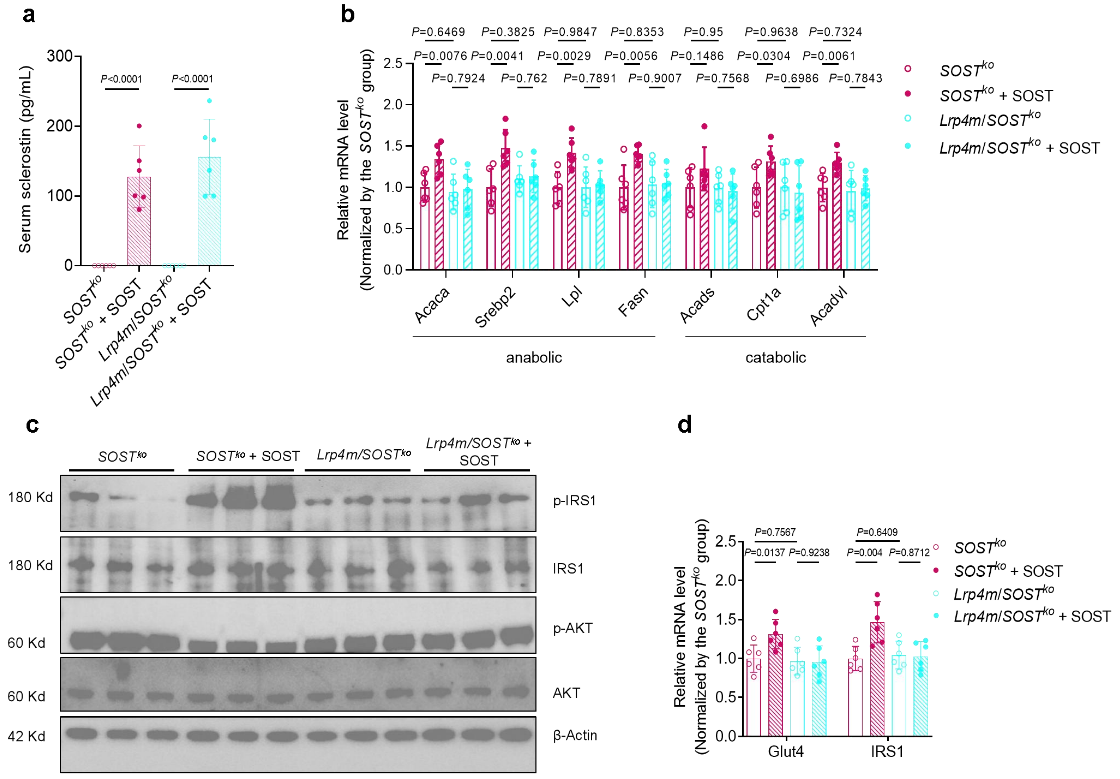

Supplementary Figure 12. The serum sclerostin levels and expression levels of genes associated with lipid and glucose metabolism in iWAT and phosphorylation levels of IRS1 and AKT in gWAT from *Lrp4m/SOST*^{ko} mice and *SOST*^{ko} mice with or without sclerostin overproduction *in vivo*. (a) Serum sclerostin levels in *Lrp4m/SOST*^{ko} mice and *SOST*^{ko} mice with or without sclerostin overproduction. (b) Expression levels of genes associated with lipid anabolism (*Acaca*, *Srebp2*, *Lpl* and *Fasn*) and catabolism (*Acads*, *Cpt1a* and *Acadvl*) in iWAT from *Lrp4m/SOST*^{ko} mice and *SOST*^{ko} mice with or without sclerostin overproduction detected by qPCR. (c) Immunoblotting of phosphorylated IRS1 and AKT in gWAT from *Lrp4m/SOST*^{ko} mice and *SOST*^{ko} mice with or without sclerostin overproduction. (d) Expression levels of genes associated with glucose metabolism in iWAT from *Lrp4m/SOST*^{ko} mice and *SOST*^{ko} mice with or without sclerostin overproduction detected by qPCR. Note: n = 6 biologically independent samples for serum sclerostin level and qPCR analysis. n = 3 biologically independent samples for western blot analysis. Statistical significance was calculated using unpaired t-test. ns: no significance. All tests were two-sided.

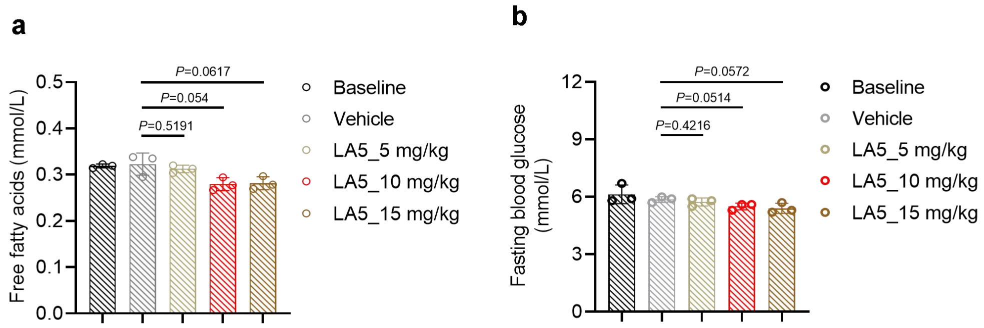

Supplementary Figure 13. The dosage optimization of blocking peptide (LA5) administration in *SOST*^{ki} mice with a small sample size. (a) Serum free fatty acids in *SOST*^{ki} mice with LA5 treatment in different dosages (5 mg/kg, 10 mg/kg, 15 mg/kg) once a day for 6 weeks. (b) Fasting blood glucose in *SOST*^{ki} mice with LA5 treatment in different dosages (5 mg/kg, 10 mg/kg, 15 mg/kg) once a day for 6 weeks. Note: All data were expressed as mean ± SD. n = 3 biologically independent samples for analysis of serum free fatty acids and fasting blood glucose. Statistical significance was calculated using unpaired t-test. All tests were two-sided.

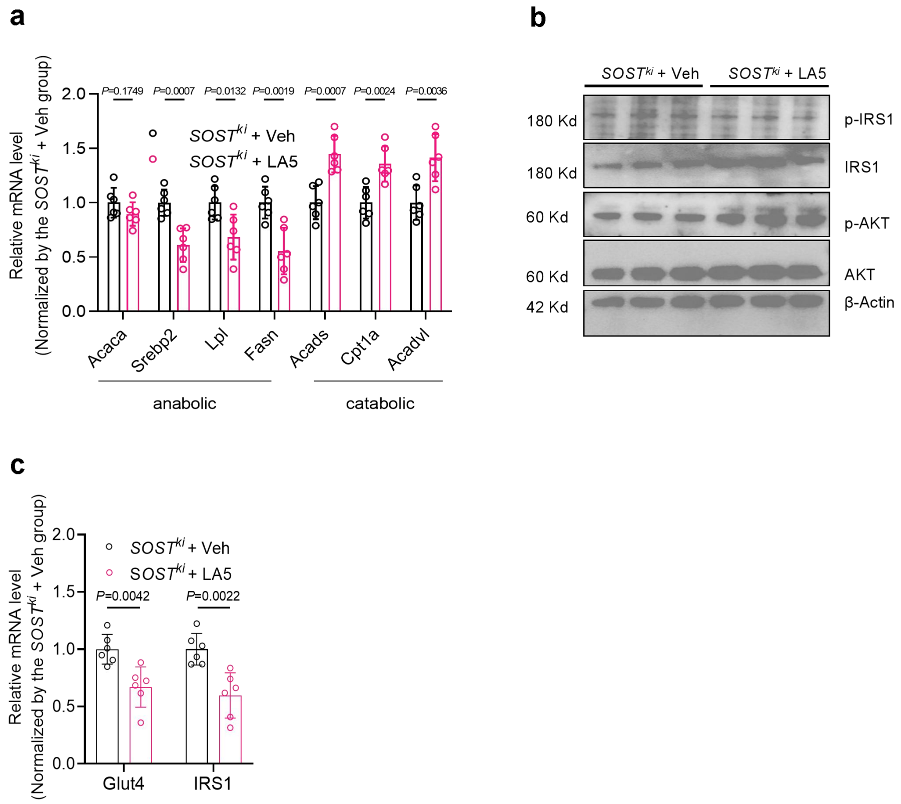

Supplementary Figure 14. The expression levels of genes associated with lipid and glucose metabolism in iWAT and phosphorylation levels of IRS1 and AKT in gWAT from *SOST*^{ki} mice with blocking peptide LA5 treatment *in vivo*. (a) Expression levels of genes associated with lipid anabolism (*Acaca*, *Srebp2*, *Lpl* and *Fasn*) and catabolism (*Acads*, *Cpt1a* and *Acadvl*) in iWAT from *SOST*^{ki} mice with or without LA5 treatment detected by qPCR. (b) Immunoblotting of phosphorylated IRS1 and AKT in gWAT from *SOST*^{ki} mice with or without LA5 treatment. (c) Expression levels of genes associated with glucose metabolism in iWAT from *SOST*^{ki} mice with or without LA5 treatment detected by qPCR. Note: n = 6 biologically independent samples for qPCR analysis. n = 3 biologically independent samples for western blot analysis. Statistical significance was calculated using unpaired t-test. All tests were two-sided.

Supplementary Table 1. The characteristics of the postmenopausal osteoporosis patients with and without type 2 diabetes mellitus.

Characteristic	POP with T2DM group	POP without T2DM group
Age (years)	65.5 ± 3.58	66.9 ± 3.19 (*P*=0.155)
FFA (mmol/L)	0.48 ± 0.19	0.37 ± 0.09 (*P*=0.0235)
Triglycerides (mmol/L)	1.59 ± 0.36	1.29 ± 0.63 (*P*=0.0479)
HbA1c (%)	8.29 ± 1.31	5.65 ± 0.39 (*P*<0.0001)
FBG (mmol/L)	6.86 ± 1.77	4.90 ± 0.37 (*P*<0.0001)

Abbreviations: POP with T2DM, postmenopausal osteoporosis patients with type 2 diabetes mellitus (n = 24); POP without T2DM, Postmenopausal osteoporosis patients without type 2 diabetes mellitus (n = 22); FFA, free fatty acids; HbA1c, glycosylated hemoglobin; FBG, fasting blood glucose. Statistical significance was calculated using unpaired t-test. All data were expressed as mean ± SD. All tests were two-sided.

Supplementary Table 2. The plasmids encoding LRP4 truncations.

Plasmid	Residues	Domains of LRP4 truncations	Molecular weight (kDa)
LRP4-FL	1-1905	Whole sequence	212
LRP4-T1	1-1725	Topological domain (Extracellular: LA 1-8, EGF like 1-3, LB 1-20, Polar residues)	192
LRP4-T2	1-1306	LA 1-8, EGF like 1-3, LB 1-15	146
LRP4-T3	1-998	LA 1-8, EGF like 1-3, LB 1-10	112
LRP4-T4	1-737	LA 1-8, EGF like 1-3, LB 1-5	82
LRP4-T5	1-434	LA 1-8, EGF like 1-2	48
LRP4-T6	1-226	LA 1-5	25
LRP4-T6_1	1-189	LA 1-4	20
LRP4-T6_2	1-146	LA 1-3	16
LRP4-T6_3	1-108	LA 1-2	11

Supplementary Table 3. The mutation strategy to obtain LRP4 muteins.

Muteins ID	Sequence of LA5 domain within LRP4 muteins
WT LRP4	PCNLEEFQCAYGRCILDIYHCDGDDDCGDWSDESDCS
LRP4-m1	AAALEEFQCAYGRCILDIYHCDGDDDCGDWSDESDCS
LRP4-m2	PCNAAAFQCAYGRCILDIYHCDGDDDCGDWSDESDCS
LRP4-m3	PCNLEEAAAAYGRCILDIYHCDGDDDCGDWSDESDCS
LRP4-m4	PCNLEEFQCAAARCILDIYHCDGDDDCGDWSDESDCS
LRP4-m5	PCNLEEFQCAYGAAALDIYHCDGDDDCGDWSDESDCS
LRP4-m6	PCNLEEFQCAYGRCIAAAYHCDGDDDCGDWSDESDCS
LRP4-m7	PCNLEEFQCAYGRCILDIAAADGDDDCGDWSDESDCS
LRP4-m8	PCNLEEFQCAYGRCILDIYHCAAADDCGDWSDESDCS
LRP4-m9	PCNLEEFQCAYGRCILDIYHCDGDAAAGDWSDESDCS
LRP4-m10	PCNLEEFQCAYGRCILDIYHCDGDDDCAAASDESDCS
LRP4-m11	PCNLEEFQCAYGRCILDIYHCDGDDDCGDWAAASDCS
LRP4-m12	PCNLEEFQCAYGRCILDIYHCDGDDDCGDWSDEAAAA
LRP4-m46	PCNLEEFQCAAARCIAAAYHCDGDDDCGDWSDESDCS
LRP4-m47	PCNLEEFQCAAARCILDIAAADGDDDCGDWSDESDCS
LRP4-m67	PCNLEEFQCAYGRCIAAAAAADGDDDCGDWSDESDCS
LRP4-m467	PCNLEEFQCAAARCIAAAAAADGDDDCGDWSDESDCS

(Note: mutated residues within LRP4 LA5 were marked in red; the rest residues within LRP4 remained unchanged.)

Supplementary Table 4. The sequences of primers.

Primer Name	Sequence (5' to 3')
Acaca-F	ATGGGCGGAATGGTCTCTTTC
Acaca-R	TGGGGACCTTGTCTTCATCAT
Acads-F	TGGCGACGGTTACACACTG
Acads-R	GTAGGCCAGGTAATCCAAGCC
Acadvl-F	CTACTGTGCTTCAGGGACAAC
Acadvl-R	CAAAGGACTTCGATTCTGCCC
ACTB-F	CCTGTGCTGCTCACCGAGG
ACTB-R	TGAAGCTGTAGCCACGCTCG
Cpt1a-F	TGGCATCATCACTGGTGTGTT
Cpt1a-R	GTCTAGGGTCCGATTGATCTTTG
Fasn-F	GGAGGTGGTGATAGCCGGTAT
Fasn-R	TGGGTAATCCATAGAGCCCAG
Glut4-F	ACACTGGTCCTAGCTGTATTCT
Glut4-R	CCAGCCACGTTGCATTGTA
IRS1-F	CGATGGCTTCTCAGACGTG
IRS1-R	CAGCCCGCTTGTTGATGTTG
Lpl-F	TTGCCCTAAGGACCCCTGAA
Lpl-R	TTGAAGTGGCAGTTAGACACAG
Srebp2-F	GCAGCAACGGGACCATTCT
Srebp2-R	CCCCATGACTAAGTCCTTCAACT
